# The Implementation of a Blended Counseling Intervention in Undergraduate Engineering Students: The Effect on Mental Health

**DOI:** 10.3390/healthcare13050511

**Published:** 2025-02-27

**Authors:** Antonios Kalamatianos, Kalliope Kounenou, Christos Pezirkianidis

**Affiliations:** 1 Department of Education, School of Education, University of Nicosia, Nicosia 2417, Cyprus; 2 Student Counseling Center, School of Pedagogical & Technological Education, 15122 Maroussi, Greece; 3 Department of Education, School of Pedagogical & Technological Education, 15122 Maroussi, Greece; kkounen@aspete.gr; 4 Laboratory of Positive Psychology, Panteion University of Social & Political Sciences, Syggrou Ave. 136, 17671 Athens, Greece; pezir@panteion.gr

**Keywords:** depression, anxiety, stress, positive psychology intervention, cognitive (psycho) therapy, group counseling

## Abstract

**Background/Objectives:** The accelerating global concern for the mental health of university students necessitates delivering intervention programs. The purpose of this study is to evaluate the effectiveness of a 5-week blended counseling group program, with two integrated approaches, positive psychology intervention and cognitive therapy, toward undergraduate engineering students’ depression, anxiety, and stress, from the Higher School of Pedagogical and Technological Education, Greece. **Methods:** Participants with a mean age of 21.12 (SD = 3.68) were administered the DASS-21. A two-way repeated-measures design was used and the two factors were condition and time. The subjects underwent two conditions, the experimental (*n* = 40), consisting of the civil and mechanical engineers, and the control (*n* = 52), that encompasses the electrical engineers. The three dependent variables, DASS-21 factors, were measured at three time points, at the beginning of the program, midway through the program, and at the end of the program. **Results:** The effect of the interaction between time and the conditions on DASS-21 depression and stress was significant. In particular, the experimental subgroups showed lower depression and stress at the end of the program in comparison with the control group. However, they did not demonstrate significantly lower scores on anxiety. Furthermore, the two intervention subgroups did not exhibit any significant mid- and post-test differences on all measures. **Conclusions:** In light of the obtained results, it can be concluded that the group counseling blended cognitive positive psychology program is an effective intervention.

## 1. Introduction

### 1.1. Student Mental Health Issues

Scholars of behavioral sciences have acknowledged that student life constitutes a stressful period of time characterized by the transition from adolescence to early adulthood. Therefore, university has been characterized by changes in course selection, major, and career choices. In this developmentally challenging phase, undergraduate students face academic evaluations and encounter, in general, new responsibilities, that may influence their mental health. They are more vulnerable to psychological distress [1,2,3,4]. The instability that they deal with may contribute to increased stress, which is known contributor to mental disorders [5]. Over 70% of students experience moderate or above moderate levels of stress and first-year students undergo severe distress [6,7,8]. Depression, anxiety, and stress have been documented as the most preponderant mental health problems among students [9] and as aggravated mental health issues during the period of COVID-19 pandemic [10]. Systematic reviews and meta-analyses have showed that, despite the alarming proportion of students reporting mental health related concerns, counselors who provide mental health care to students are overwhelmed and fail to meet the needs of all the students [11,12]. This demonstrates the need for further organized interventions in the tertiary-level education. Psychoeducational counseling groups have been the most common group interventions offered in universities, they are focused, time-limited, process-oriented and structured topic, they personalize the information, teach skills, and balance content with discussion [13]. Additionally, brief interventions have shown useful results in responding to the mental health needs in educational settings and the reduction in depression, anxiety, and stress [14,15].

### 1.2. Positive Psychology Interventions to Students

A practical response to the aforementioned mental health problems is to enhance students’ mental well-being through the services offered by the counseling centers. Positive psychology paradigm generally encourages people to work with inner strengths and promotes flourishing [16]. Positive psychology interventions (PPI) constitute strategies that emphasize on increasing happiness, well-being, and positive cognitions [17]. The results suggest that these interventions are profitable for university students in increasing their well-being and decreasing anxiety and depression [18,19,20] and reducing their perceived stress [21]. A six-week psychoeducational multicomponent PPI program, implemented to Greek young adults, was effective in decreasing levels of depression, anxiety, and stress [22]. Positive psychology interventions have also been applied in Greek primary educational settings with beneficial outcome in promoting well-being, enhancing positive emotion and reducing trait and state anxiety [23,24], as well as in Greek first-year psychology students with significant decreases in depression, anxiety, and stress [25].

### 1.3. Cognitive Therapy Interventions to Students

Moreover, cognitive behavior therapy (CBT) is a widely applied approach which is based on the idea that cognitions and behaviors affect feelings and which helps people face problems by altering one’s thoughts and behaviors [26]. CBT has been documented as an effective intervention in reducing depression in clinical settings in a group format and as a cost-effective treatment approach whose potential for benefits significantly outweighs the cost [27]. Research has also displayed the effectiveness of group CBT in treating anxiety [28,29], and stress in students [30]. It has also been significantly associated with positive outcomes for mental health and well-being, as well as with reducing trait anxiety and dysphoric mood states [31], depression in undergraduate students [32], and stress in students in group format [33].

### 1.4. Integration of Cognitive and Positive Psychology Interventions

In the last decade there have been attempts to integrate traditional CBT elements with positive psychology interventions, such as Fava’s well-being therapy that aimed at eradicating cognitive barriers to well-being [34]. Bannink [35] also introduced positive CBT that combined CBT with a focus on positive characteristics and mental health. Positive psychology approaches share conceptual overlap with CBT interventions. In particular, focus on the here and now, cognitive reappraisal, and treating the client as collaborative partner are some principles found in both rationales [36]. However, there are points of difference and, consequently, the integration of positive aspects of psychological functioning to cognitive treatment and the use of the students’ strengths may help them reduce their clinical difficulties [37]. Research has suggested that the association of the two therapeutic strategies could improve the usefulness of interventions [37].

Numerous studies have demonstrated that blended interventions can improve outcomes for people dealing with depression, anxiety, and stress, even on a long-term basis, while also embracing gratitude, fostering well-being, and boosting strengths [38,39,40]. Further, in a qualitative study by Geschwind et al. [41] it was noticed that patients with major depressive disorder preferred positive CBT compared to traditional one and attributed their choice to several reasons, such as (re)discovering optimism as a personal strength. Analyses have also indicated that positive CBT, compared with traditional one, provided more statistically significant and clinically relevant changes in depression and negative affect [42]. Other research [43] has showed that stress was significantly reduced in university students following CBT combined with gratitude based positive psychology intervention. It has also been suggested that the augmentation of CBT’s efficacy may be attained by focusing on positive mental health and including themes, such as strengths and life-goals [44] and by emphasizing positive features [45] and well-being [46].

### 1.5. The Purpose of the Present Study

The main objective of the present study was firstly to examine whether a brief blended cognitive treatment and positive psychology group intervention could be effective in reducing depression, anxiety, and stress scores in university students; and secondly to compare the two processes. Groups may constitute an effective setting for evaluating dysfunctional cognitions and envisioning alternative ones, practicing positive skills, and changing impaired intrapersonal and interpersonal patterns [47]. The efficacy of PPI has been compared to available empirically based treatments, such as CBT, with adults and university students, aiming to accomplish various goals, for instance to treat depression and enhance psychological well-being [48]. Nevertheless, to the best of our knowledge, there has not been any systematic survey to investigate the combination of these two interventions, in a short-term group program, to Greek undergraduate students, especially engineers. We consider the content of the paper significant, since we implemented a not-often-used blended cognitive and positive psychology intervention, combining techniques from distinct theoretical trajectories that focus on different areas, such as modifying thought patterns and enhancing well-being, in order to promote mental health and, thus, allowing for a holistic view of both reducing negative symptoms and strengthening personal growth, consequently complementary techniques. Moreover, the current research is timely and important, because dropout, globally and within Greece, affects all higher education institutions, especially in STEM (Science, Technology, Engineering, and Mathematics) fields [49]. Improved mental health and counseling services have been proposed as ways of addressing this challenge and preventing dropout [50].

Thus, along with the background of previous similar work with adults, the following questions constitute the research agenda of the present study:

RQ1. Will the two experimental subgroups, civil and mechanical engineers, demonstrate lower score than the control group, the electrical engineers, in all scales, depression, anxiety, and stress, in mid-test and post-test assessments?

RQ2. Will the two psychological interventions have a different impact on the two experimental subgroups?

## 2. Materials and Methods

### 2.1. Study Sample

The participants (*N* = 92) were 66 male students engineering educators (71.7%) and 26 female (28.3%) of the Higher School of Pedagogical and Technological Education (aspete), Greece, with average age of 21.12, SD = 3.68. The experimental group consisted of 40 students, 23 mechanical engineers and 17 civil engineers, 25 male (62.5%) and 15 female (37.5%), mean age = 21.25, SD = 4.93. The control group included 52 electrical engineers, 41 male (78.8%) and 11 female (21.2%), mean age = 21.02, SD = 2.36. Five students from the experimental group and 14 from the control group failed to fully attend the program or were lost to post-test. Among all the participants, 20 students (21.7%) reported having selected aspete as their first choice for the national entrance exams to universities and 72 (78.3%) as a later choice. With regard to learning difficulties, 22 (23.9%) students had diagnosis and 70 (76.1%) did not. Finally, 33 (82.5%) engineers in the experimental group were 2nd-year students, while 5 (12.5%) were 3rd year, and 2 (5%) 5th year; whereas 49 (94.23%) participants in the control group were 3rd-year students, 1 (1.92%) was 5th year, and 2 (3.85%) were graduates.

### 2.2. Survey Tools

We used an improvised basic demographic questionnaire that included general background information on gender, age, faculty, learning difficulties diagnosis, and selection order of schools for the national entrance exams to higher education institutions. A supplementary improvised student satisfaction questionnaire was also administered and included items rated on a 5-point Likert scale, 1 = very disappointed, 5 = very satisfied, about teaching quality, teachers’ knowledge, class organization, university notes, testing and grading mode, for example assignments, written exams, etc., administrative assistance, department’s e-platform, and technical services.

In addition, all participants were asked to fill the Depression, Anxiety, Stress Scales-21 (DASS-21). It is the short form of the 42-item Depression, Anxiety, Stress Scales [51], divided into three self-report scales and designed to measure the emotional states of depression, anxiety, and stress. Each scale contains 7 items. Each scale’s items are rated on a 4-point Likert scale, 0 = did not apply to me at all, 3 = applied to me very much or most of the time. The three scales evaluate the individual’s dysphoria, self-deprecation, lack of interest, hopelessness, etc., one’s situational anxiety, subjective experience of anxious affect, etc., and one’s difficulty relaxing, being easily upset, persistent arousal, etc., respectively. It is based on a dimensional conception of mental disorder and was developed on the idea that there are differences of degree in its three factors between patients and normal subjects and subsequently cannot be used for the allocation of patients to distinct diagnostic categories. DASS-21 possesses satisfactory psychometric properties, good internal consistency ranging between 0.70 and 0.94 for the three scales, in clinical samples and normal subjects, and acceptable convergent and discriminant validity discriminating adequately between clinical and non-clinical populations, and can be used as a reliable and valid instrument in the Greek population [52]. In the present study, DASS-21 had good reliability in all three domains of depression (a = 0.83), anxiety (a = 0.78), and stress (a = 0.83) and overall (a = 0.91).

### 2.3. Ethics

The study protocol was approved by the Faculty Ethics Committee of the Higher School of Pedagogical and Technological Education, Greece (protocol number: 1/4-4-2023) and research was conducted following the Declaration of Helsinki’s ethical principles. All participants took part in the research on a voluntary basis and did not receive any reward for their participation. They were informed on the confidentiality of their responses, then they filled in a written consent form, and subsequently, the questionnaires were administered.

### 2.4. Procedure

The sample was collected through convenience sampling technique. The psychoeducational program was implemented by the psychologist of the counseling center of the School of Pedagogical and Technological Education (aspete), Greece. We gathered 92 subjects, of whom 40 performed two intensive short-term interventions, a cognitive psychotherapy and a positive psychology one, which were counterbalanced in the two experimental subgroups whilst in the control trial, 52 subjects continued their normal academic activities without any intervention. Counseling consisted of 5 weekly 2-h sessions.

The first experimental subgroup of civil engineers received a positive psychology intervention for 2½ weeks. In the beginning psychoeducation regarding the main characteristics of positive psychology was offered along with related handouts. Subsequently, techniques were drawn from the 8-week positive psychotherapy group by Parks and Seligman [53] and in particular the Three Good Things and exercises and assignments related with gratitude and character strengths. A literature review [25] has pointed out that these were among the most commonly used and empirically based interventions.

Gratitude, being thankful for the good things, is a crucial factor of positive psychology. The positive effect of gratitude interventions in students has been indicated in previous research [54]. We used two categories of gratitude techniques, firstly the self-reflective practice of writing a gratitude journal used as a tool for self-expression; secondly, the interactive method of active expression of gratitude to others and giving small tokens of appreciation by writing a letter of gratitude. In addition, strength refers to internal capacities and values [55] and character strengths are the psychological ingredients that define the virtues [56]. Exercises were included so that the students could identify their positive traits and use them to improve and maintain increased levels of well-being. Studies have illustrated the positive effect of acknowledging the character strengths in reducing symptoms of depression, anxiety, and stress [38,57].

On the other hand, the second subgroup of mechanical engineers entailed cognitive psychotherapy for 2½ weeks; at the beginning, psychoeducation regarding the basic principles of cognitive therapy together with related handouts, then finding process errors in thinking and matching them with negative thoughts related to university activities, identifying and exorcising negative thinking patterns, and challenging and modifying automatic negative thoughts. Homework assignments were introduced because their use in order to disconfirm dysfunctional thinking patterns and to develop more functional responses is considered to be a main ingredient of CBT [58]. Thus, we focused on monitoring and disconfirming or changing unrealistically negative automatic thoughts and adopting more functional responses.

In the second half of the program, the civil engineers were delivered the cognitive therapy intervention and the mechanical engineers, vice versa, attended the positive psychology sessions (see Table 1). At the start of each session the summary of the key ideas was provided. Then, between-session homework was reviewed and students were encouraged to ask questions, give feedback, and participate, generally, in discussions during sessions. Leaflets with information on each approach, with between meetings assignments, and worksheets were handed at each session. Subsequently, the topic of the week was introduced. At the end of each meeting, homework exercises tailored to the students’ needs were assigned.

### 2.5. Design and Statistical Analysis

The statistical analysis was carried out with SPSS 28. Initially, chi-square test of independence was used to investigate differences between groups in baseline demographic variables. Independent *t*-tests were used to trace differences between the intervention and the control group in the supplementary questionnaire. Repeated-measures ANCOVAs controlling for the baseline scores of all DASS-21 factors were performed. We also used a two-way repeated-measures ANOVA and measured the students’ scores at depression, anxiety and stress (dependent variable), over three time points, namely when subjects underwent three conditions, the two factors (independent variables) being “time, that is pre-intervention, middle of intervention, and post-intervention” and “conditions, videlicet intervention group, especially mechanical and civil engineering educators, and control group, particularly electrical engineering educators”. We aimed to understand if there is an interaction between the two factors on the students’ scores at depression, anxiety, and stress. We compared the mean differences between groups that have been split on these two within-subjects factors. Finally, we examined the presence of the two intervention subgroups’ differences in the medium and post-phases measures of depression, anxiety, and stress through independent *t*-tests. We used an alpha level of 0.05 for all statistical tests.

## 3. Results

### 3.1. Independent t-Tests Comparing the Intervention Group and the Control Group on Student Satisfaction Questionnaire Items

The independent *t*-tests indicated that the intervention group in comparison with the control group showed greater satisfaction toward testing and grading mode. The means and standard deviations for the student satisfaction with university services’ items are presented in Table 2.

### 3.2. Two-Way Repeated-Measures ANOVAs

Normality data check, with the use of Q–Q plots, skewness and kurtosis values, as well as z-scores ±3.29 [59,60], concluded that the distribution of the sample was normal. We also found no significant association between group and gender, χ*χ*^2^ (1, *N* = 92) = 2.98, *p* = 0.084. Moreover, the participants of the two groups did not differ by whether aspete was the first choice for university entrance exams or not, χ^2^ (1, *N* = 92) = 1.38, *p* = 0.24, and by the presence or absence of learning difficulties diagnosis, χ^2^ (1, *N* = 92) = 0.50, *p* = 0.479. We noted significant association between group and year of studies χ^2^ (1, *N* = 92) = 70.825, *p* < 0.001, Φ = 0.877. A two-way repeated-measures ANOVA was performed to evaluate the effect of time on DASS-21 depression, anxiety, and stress. The means and standard deviations for depression, anxiety, and stress are presented in Table 3. Initially we conducted repeated-measures ANCOVAs controlling for the baseline scores of all DASS-21 variables. We found for the two experimental subgroups and the control group that the difference in the depression and stress scores, from mid-test to post-test, showed a significant interaction with the pre-test depression and stress scores correspondingly, *F* (1, 0.253) = 5.805, *p* < 0.05, partial η^2^ = 0.062, *F* (1, 0.846) = 10.911, *p* < 0.01, partial η^2^ = 0.110, whereas a not significant interaction in the case of anxiety, *F* (1, 0.008) = 0.254, *p* = 0.62, partial η^2^ = 0.003, was discovered. Thus, the following results, as for depression and stress, should be interpreted with caution.

#### 3.2.1. DASS-21 Depression

1. Whole sample

**Figure 1:**
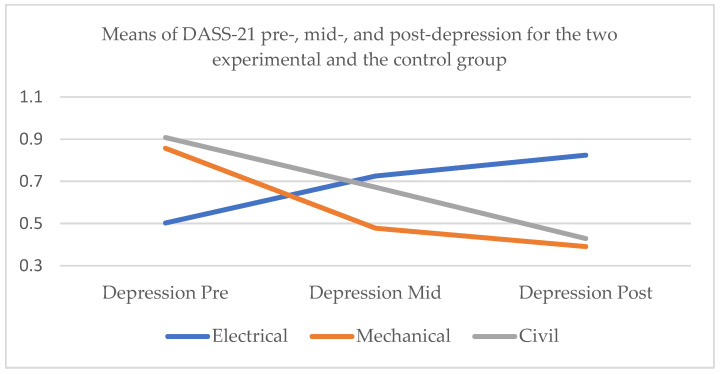
Means of DASS-21 pre-, mid-, and post-depression for the two experimental subgroups, the mechanical and civil engineers, and the control group, the electrical engineers.

Firstly, with relation to depression, for the whole sample, Mauchly’s test indicated that the assumption of sphericity had been violated, χ^2^ = 14.707, *p* < 0.01, and therefore degrees of freedom were corrected using Huynh–Feldt estimates of sphericity (ε = 0.902). The effect of the factors time × conditions on DASS-21 depression was significant at the 0.05 level, *F* (3.609, 160.586) = 27.075, *p* < 0.001, partial η^2^ = 0.378. Post hoc pairwise comparisons with a Bonferroni adjustment indicated that there was significant difference in depression at the initial assessment and the mid-test (*p* < 0.05). Similarly, there was significant difference between the depression scores at pre-test and post-test (*p* < 0.001), but no significant difference between the depression at mid-test and post-test (*p* = 0.092) (Figure 1).

2. Electrical engineers

As for the control group, Mauchly’s test indicated that the assumption of sphericity had been violated, χ^2^ = 47.237, *p* < 0.001, and therefore degrees of freedom were corrected using Greenhouse–Geisser estimates of sphericity (ε = 0.621). The effect of time on DASS-21 depression was significant at the 0.05 level, *F* (1.241, 63.306) = 19.830, *p* < 0.001, partial η^2^ = 0.280. Post hoc pairwise comparisons with a Bonferroni adjustment indicated that depression was significantly higher at the mid-test than at the initial assessment (*p* < 0.01), at the post-test than the pre-test (*p* < 0.001), and at the post-test than the mid-test (*p* < 0.01).

3. Mechanical engineers

As for the mechanical engineers, Mauchly’s test indicated that the assumption of sphericity had been violated, χ^2^ = 7.596, *p* < 0.05, and therefore degrees of freedom were corrected using Huynh–Feldt estimates of sphericity (ε = 0.813). The effect of time on DASS-21 depression was significant at the 0.05 level, *F* (1.627, 35.784) = 22.754, *p* < 0.001, partial η^2^ = 0.508. Post hoc pairwise comparisons with a Bonferroni adjustment indicated that depression was significantly lower at the mid-test than at the initial assessment (*p* < 0.001) and at the post-test than the pre-test (*p* < 0.001). However, there was no significant difference between the depression scores at the post-test and the mid-test (*p* = 0.985).

4. Civil engineers

As for the civil engineers, Mauchly’s test indicated that the assumption of sphericity had been violated, χ^2^ = 14.913, *p* < 0.01, and therefore degrees of freedom were corrected using Greenhouse–Geisser estimates of sphericity (ε = 0.614). The effect of time on DASS-21 depression was significant at the 0.05 level, *F* (1.227, 19.632) = 12.697, *p* < 0.01, partial η^2^ = 0.442. Post hoc pairwise comparisons with a Bonferroni adjustment indicated that depression was significant lower at the mid-test than the initial assessment (*p* < 0.01) and at the post-test than the pre-test (*p* < 0.01), but not significantly lower at the post-test than the mid-test (*p* = 0.081).

#### 3.2.2. DASS-21 Anxiety

1. Whole sample

**Figure 2:**
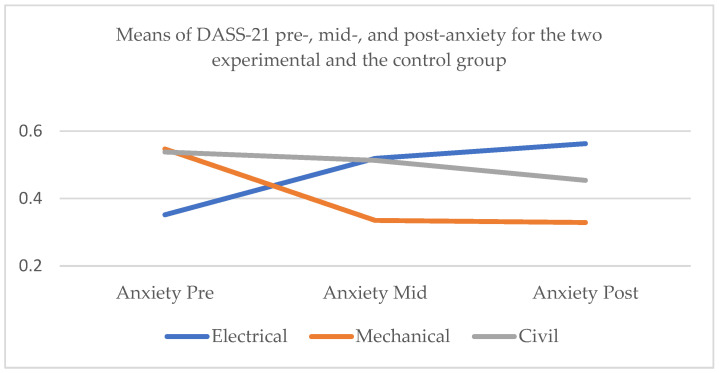
Means of DASS-21 pre-, mid-, and post-anxiety for the two experimental subgroups, the mechanical and civil engineers, and the control group, the electrical engineers.

Secondly, with respect to anxiety for the whole sample, Mauchly’s test indicated that the assumption of sphericity had been violated, χ^2^ = 14.273, *p* < 0.01, and therefore degrees of freedom were corrected using Huynh–Feldt estimates of sphericity (ε = 0.906). The effect of the factors time ×conditions on DASS-21 anxiety was significant at the 0.05 level, *F* (3.622, 161.189) = 10.521, *p* < 0.001, partial η^2^ = 0.191. Post hoc pairwise comparisons with a Bonferroni adjustment indicated that there was no significant difference in anxiety at the initial assessment and the mid-test (*p* = 1.000). Similarly, there was no significant difference between the anxiety scores at pre-test and post-test (*p* = 1.000) and at mid-test and post-test (*p* = 1.000) (Figure 2).

2. Electrical engineers

As for the control group, Mauchly’s test indicated that the assumption of sphericity had been violated, χ^2^ = 48.819, *p* < 0.001, and therefore degrees of freedom were corrected using Greenhouse–Geisser estimates of sphericity (ε = 0.613). The effect of time on DASS-21 anxiety was significant at the 0.05 level, *F* (1.226, 62.547) = 13.969, *p* < 0.001, partial η^2^ = 0.215. Post hoc pairwise comparisons with a Bonferroni adjustment indicated that anxiety was significantly higher at the mid-test than at the initial assessment (*p* < 0.01) and at the post-test than the pre-test (*p* < 0.001). However, there was no significant difference between the anxiety scores at the post-test and the mid-test (*p* = 0.103).

3. Mechanical engineers

As for the mechanical engineers, Mauchly’s test indicated that the assumption of sphericity had been violated, χ^2^ = 21.512, *p* < 0.001, and therefore degrees of freedom were corrected using Greenhouse–Geisser estimates of sphericity (ε = 0.609). The effect of time on DASS-21 anxiety was significant at the 0.05 level, *F* (1.219, 26.813) = 5.690, *p* < 0.05, partial η^2^ = 0.205. Post hoc pairwise comparisons with a Bonferroni adjustment indicated that anxiety was significantly lower at the mid-test than at the initial assessment (*p* < 0.001). However, there was no significant difference between the anxiety scores at the post-test and the pre-test (*p* = 0.072) and at the post-test and the mid-test (*p* = 1.000).

4. Civil engineers

As for the civil engineers, Mauchly’s test indicated that the assumption of sphericity had been violated, χ^2^ = 35.440, *p* < 0.001, and therefore degrees of freedom were corrected using Greenhouse–Geisser estimates of sphericity (ε = 0.525). The effect of time on DASS-21 anxiety was not significant at the 0.05 level, *F* (1.049, 16.791) = 1.343, *p* = 0.265, partial η^2^ = 0.077. Post hoc pairwise comparisons with a Bonferroni adjustment indicated that there was no significant difference between the anxiety scores at the mid-test and at the initial assessment (*p* = 0.248), at the post-test and the pre-test (*p* = 0.687), and at the post-test and the mid-test (*p* = 1.000).

#### 3.2.3. DASS-21 Stress

1. Whole sample

**Figure 3:**
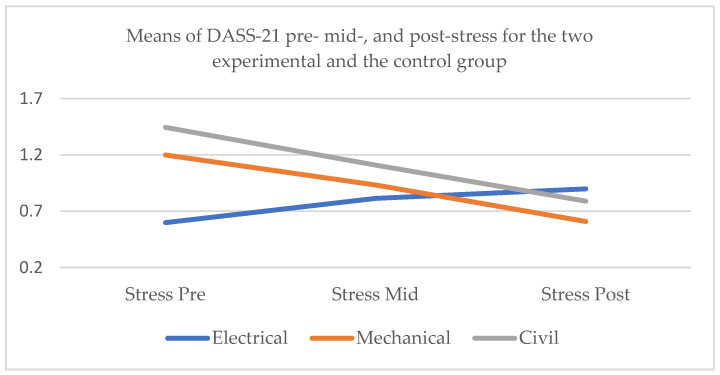
Means of DASS-21 pre- mid-, and post-stress for the two experimental subgroups, the mechanical and civil engineers, and the control group, the electrical engineers.

Thirdly, with respect to stress for the whole sample, Mauchly’s test indicated that the assumption of sphericity had been violated, χ^2^ = 21.570, *p* < 0.001, and therefore degrees of freedom were corrected using Huynh–Feldt estimates of sphericity (ε = 0.854). The effect of the factors time × conditions on DASS-21 stress was significant at the 0.05 level, *F* (3.415, 151.948) = 22.222, *p* < 0.001, partial η^2^ = 0.333. Post hoc pairwise comparisons with a Bonferroni adjustment indicated that there was significant difference in stress at the initial assessment and the mid-test (*p* < 0.05). Similarly, there was significant difference between the stress scores at pre-test and post-test (*p* < 0.001) and at mid-test and post-test (*p* < 0.01) (Figure 3).

2. Electrical engineers

As for the control group, Mauchly’s test indicated that the assumption of sphericity had been violated, χ^2^ = 23.847, *p* < 0.001, and therefore degrees of freedom were corrected using Greenhouse–Geisser estimates of sphericity (ε = 0.725). The effect of time on DASS-21 stress was significant at the 0.05 level, *F* (1.450, 73.950) = 13.470, *p* < 0.001, partial η^2^ = 0.209. Post hoc pairwise comparisons with a Bonferroni adjustment indicated that stress was significantly higher at the mid-test than at the initial assessment (*p* < 0.01) and at the post-test than the pre-test (*p* < 0.001). However, there was no significant difference between the stress scores at the post-test and the mid-test (*p* = 0.075).

3. Mechanical engineers

As for the mechanical engineers, Mauchly’s test indicated that the assumption of sphericity had been violated, χ^2^ = 27.875, *p* < 0.001, and therefore degrees of freedom were corrected using Greenhouse–Geisser estimates of sphericity (ε = 0.576). The effect of time on DASS-21 stress was significant at the 0.05 level, *F* (1.153, 25.363) = 12.992, *p* < 0.01, partial η^2^ = 0.371. Post hoc pairwise comparisons with a Bonferroni adjustment indicated that stress was significantly lower at the mid-test than at the initial assessment (*p* < 0.001) and at the post-test than the pre-test (*p* < 0.01). However, there was no significant difference between the stress scores at the post-test and the mid-test (*p* = 0.057).

4. Civil engineers

As for the civil engineers, Mauchly’s test indicated that the assumption of sphericity had been violated, χ^2^ = 13.974, *p* < 0.01, and therefore degrees of freedom were corrected using Greenhouse–Geisser estimates of sphericity (ε = 0.623). The effect of time on DASS-21 stress was significant at the 0.05 level, *F* (1.245, 19.924) = 19.390, *p* < 0.001, partial η^2^ = 0.548. Post hoc pairwise comparisons with a Bonferroni adjustment indicated that stress was significantly lower at the mid-test than at the initial assessment (*p* < 0.001), at the post-test than the pre-test (*p* < 0.001), and at the post-test than the mid-test (*p* < 0.05).

### 3.3. Independent t-Tests Comparing the Two Experimental Subgroups on DASS-21 Variables

The independent *t*-tests indicated that there were no significant differences between the two experimental groups, mechanical and civil engineers, in DASS-21 depression scores, mid, *t* (38) = −1.094, *p* = 0.281, and post, *t* (38) = −0.261, *p* = 0.795, in anxiety scores, mid, *t* (38) = −1.237, *p* = 0.224, and post, *t* (38) = −0.915, *p* = 0.366, and finally in stress scores, mid, *t* (38) = −1.042, *p* = 0.304, and post, *t* (38) = −1.231, *p* = 0.226.

## 4. Discussion

The aim of this study was to blend cognitive and positive psychology intervention strategies within a single short-term counseling program implemented to university students. Our findings point out that the first research question was supported; participants in the group counseling condition, compared with those in the control condition, showed greater mid-test and post-test improvement, namely significantly mid- and post-test lower scores, in DASS-21 stress and significantly mid- and post-test, compared to baseline, lower scores, in DASS-21 depression, but not significantly lower scores in DASS-21 anxiety. Our results are consistent with previous studies; for instance, the application of character strengths in psychological interventions that also focus on promoting virtues such as gratitude, alongside cognitive techniques that challenge negative thinking patterns, have also been found effective [61]. Anyway, the gratitude intervention has managed to reduce negative affect and depression symptoms [54]. Contrary to earlier studies [25], we discovered no significant differences in anxiety between the experimental and the control group. The lack of statistical significance in the students’ anxiety scores may be due to the program’s failure to address specific mental health issues related to autonomic arousal and the subjective experience of anxious affect. Overall, this study constitutes another empirical argument in favor of promoting interventions that integrate positive functioning in mental health programs that focus on decreasing clinical symptoms.

With respect to depression and stress, although the two conditions had significant baseline differences, which fact compels us to be rather parsimonious with our conclusions, the control group’s depression and stress scores would increase, regardless of the intervention. This may be due to the approaching exam time or to the groups’ year of studies, since the electrical engineering educators were, compared to the other students, in their majority, in a later year of their studies. They were mostly 3rd-year students and had started their academic career in the second year of COVID-19, with distance education, unlike the experimental groups that started their studies one year later. The results pointed out that the electrical engineers felt more disappointed with teaching quality, the knowledge their teachers demonstrated, the notes distributed by the university to the students, and the testing and grading mode, that is, the use of assignments, oral, written or distance exams.

With regard to the second research question, the present study also revealed no significant differences between the two experimental subgroups in any DASS-21 variable, indicating that both civil and mechanical engineers were approximately benefited to the same extent by the intervention. Similarly, no differences in reducing depression and negative affect emerged between 10-session PPI and CBT protocols delivered to women [37]. This lack of significant differences is a common finding in meta-analytic studies, when researchers compare modalities in well-planned and executed interventions for different populations with depression and in different settings [62,63,64]. The two intervention subgroups showed significant changes in depression and stress in the first period of the program and significant or marginally insignificant decreases in the second half of it, maybe due to the decelerating curve of improvement in psychotherapy that could be associated with the increasing difficulty of the program’s goals over its course [64].

As for limitations of the study, we should mention that the sample size was small, the study design did not include a randomized controlled trial, mediational psychological factors were not examined, and the measurement through self-report questionnaire with Likert-scale is susceptible to response biases, such as social desirability. Furthermore, we utilized convenience sampling which limits the generalizability of the results. Additionally, we did not include the behavioral dimension of CBT that can help participants become exposed to their own negative emotions due to practical difficulties related to the students’ academic program. Hence, we did not use techniques involving behavioral experiments in order to determine the truth of students’ beliefs by experimenting with their negative beliefs in real life. Cognitive distortions and negative automatic thoughts were briefly processed without addressing assumptions, evaluations, and core beliefs; consequently, further evidence is needed.

With regard to advantages of the study, there seems to be increased cost-effectiveness of providing counseling to students in groups rather than individually. Counselors can work with more people in one session than with individual ones. Needless to say, many participants find it useful to meet others who face similar difficulties; they may also have the opportunity to learn from the homework tasks and generally learn from or more easily share their experiences to the other group members, receive help by others, view others’ opinion as more neutral than the opinion of the counselor, and consequently be more open to cognitive challenging [65]. Further, it seems that the two combined interventions were adequately promoted and well marketed to the students with relatable to them messaging, resulting in the students feeling motivated to take part in the intervention and engaging with the group program. Taking into account that the engineering educators’ willingness and motivation to participate in research are not high, since despite their elevated stress they are unwilling to seek professional help [66] and tend to trivialize and normalize the stress challenges [67], it becomes apparent that an intervention that focuses on promoting positive thinking patterns, especially in engineering students, is necessary and beneficial.

The results of the present study have additional practical implications. To our knowledge the present study is the first attempt to explore the effectiveness of a combination of two therapeutic modalities in improving the mental health of Greek engineering students. If we consider that academic procrastination is a very common behavior linked with depression, anxiety, and stress [68], and that interruption and academic dropout are international phenomena, associated with depression, which impact the students and their families, and particularly acute within technology and engineering, we can realize why interventions supporting student completion seem to be of paramount importance [69,70]. Furthermore, students who need opportunities to build new skills and increase their well-being and who at the same time struggle with academic demands and life transitions may benefit from group psychoeducational counseling programs, even short-term. Instead of languishing on a waitlist for an individual session, students can have rapid access to group sessions in order to face their mental health issues. Mental health practitioners and university counseling center psychologists could design and apply cost-effective interventions that focus on highlighting positive attributes, managing anxiety and stress, promoting positive thinking patterns, improving coping strategies, and enhancing student engagement. It seems that Greek universities could discover ways to incorporate a broader culture of wellness into their policies.

In view of the limitations, we can put forward suggestions for future research. The study would benefit from replication with the use of randomization, which may also increase the generalizability of the results, from greater sample, long-term intervention, and follow-up assessment. The interventions need ongoing reinforcement and sustained support. Without proper support after the end of a group program, students may revert to old thought patterns. Follow-up sessions can help ensure that students maintain progress. The fact that no significant differences between the two intervention modalities were found should not prevent research replication because long-term effects could be different. Long-term studies may give the opportunity to discover patterns of change in distress and how they affect the therapeutic outcome [64]. Brief CBT consists of material compressed into four to eight sessions with one or two follow-ups occurring at post-intervention increasing intervals, two to four weeks after the termination [71]. Additionally, recent metanalysis of PPIs studies with clinical and non-clinical child and adult populations in 41 countries included an average of ten sessions over six weeks offered in multiple formats and contexts, aiming at depression, anxiety, and stress, and measuring gains maintenance at three months follow-up [18]. Another metanalysis examining the effect of CBT for depression among university students found that the effect was largely maintained at six months but not at 12 months follow-up [72]. Therefore, brief blended group programs of longer duration and follow-ups during at least two academic semesters could be implemented and help determine whether they would produce long-term effects. Furthermore, the efficacy of a brief blended group intervention could be compared to empirically-based, traditional therapeutic modalities of longer duration and investigate whether the former would produce a clinically, equally significant change or not in depression, anxiety, and stress with the latter. The findings of metanalyses [39,73] have also suggested that PPIs for treating depressed people should be delivered for longer periods of time and at individual therapy. However, a recent metanalysis [74] has revealed that the evidence for the differences between shorter versus long-term psychotherapies for mood and anxiety disorders remains unclear. Additionally, future studies could simultaneously examine whether significant differences would emerge when comparing positive CBT with CBT and PPI in reducing depression, anxiety, and stress and improving positive affectivity and emotional well-being. Lastly, future research should not be limited to samples of psychology students only, because this may limit the external validity of the findings [75]. On the contrary, it should be conducted with students from other faculties, with high dropout rates, especially those studying technology and engineering [76,77].

In addition, multicomponent programs have proved to be beneficial [78,79,80] for depression, anxiety, and stress. Thus, it is recommended that other positive psychology techniques be utilized in future blended group programs, for instance, strengths interviews, the three blessings exercise and practicing optimistic attribution [81,82,83] so as to focus on and stimulate positive emotions, which positive psychology feature was not integrated in our program. In that way, we may examine the potential impact of diverse factors and methods and understand their interconnectedness and contribution to the therapeutic result [75]. Further cognitive as well as behavioral techniques can be integrated, for example problem-solving skills, the combination of downward and upward techniques, that focus on the positive reactions to a given situation, and behavioral experiments, for example the experimental manipulation of the environment [84]. Lastly, forthcoming research could also explore the combined benefits of the integration of cognitive behavioral therapy with principles of positive psychology and its effectiveness in addressing various academic-related conditions, such as procrastination, academic efficacy, career development, and meaning in life.

## 5. Conclusions

The primary goal of the present study was to examine whether a 5-week psychoeducational, cognitive positive psychology blended counseling program can evoke a decrease in depression, anxiety, and stress. By focusing on enhancing well-being and cognitive restructuring, this program can be effective in alleviating anxiety and mostly depression and stress.

## Figures and Tables

**Table 1:** Description of the blended group program in every session.

Session Civil Engineers	Topic	Content	Tasks/Exercises	Homework
1	Initial measurement—introduction in positive psychology	Administration of questionnaires, information about the goal of the intervention—aim of the Three Good Things	Three Good Things	Three good things every day in a weekly span
2	Character strengths, gratitude	Presentation of virtues and character strengths, theory about gratitude	Everyday use of character strengths and new ways of application, future goals and character strengths that can help achieve them	List of accomplishments and achievements, journal of gratitude
3	Gratitude—middle measurement—introduction in cognitive psychotherapy	Theory about gratitude and its benefits—administration of questionnaires—theory about the errors in thinking	Letter of gratitude, personal motto—examples of cognitive distortions	Matching types of cognitive distortions with their examples
4	Recognizing negative automatic thoughts	Theory about negative thoughts and ways of discovering them	Identifying cognitive distortions and associating them with provoking situations	Journal of negative automatic thoughts
5	Dismantling negative automatic thoughts—ending and final measurement	Theory about challenging negative thoughts and finding alternatives—administration of questionnaires	Challenging negative automatic thinking and reframing it into alternative rational one, personal motto	
**Session Mechanical Engineers**				
1	Initial measurement—introduction in cognitive psychotherapy	Administration of questionnaires—information about the goal of the intervention—theory about the errors in thinking	Examples of cognitive distortions	Matching types of cognitive distortions with their examples
2	Recognizing negative automatic thoughts	Theory about negative thoughts and ways of discovering them	Identifying cognitive distortions and associating them with provoking situations	Journal of negative automatic thoughts
3	Dismantling negative automatic thoughts—middle measurement—introduction in positive psychology	Theory about challenging negative thoughts and finding alternatives—administration of questionnaires—aim of the Three Good Things	Challenging negative automatic thinking and reframing it into alternative rational one, personal motto—Three Good Things	Three good things every day in a weekly span
4	Character strengths, gratitude	Presentation of virtues and character strengths, theory about gratitude	Everyday use of character strengths and new ways of application, future goals and character strengths that can help achieve them	List of accomplishments and achievements, journal of gratitude
5	Gratitude—ending and final measurement	Theory about gratitude and its benefits—administration of questionnaires	Letter of gratitude, personal motto	

**Table 2:** Differences between the experimental group and the control group in student satisfaction with university services.

Student Satisfaction with University Services Items	Experimental		Control		df	*t*	*p*
	M	SD	M	SD			
Class organization	2.85	1.099	2.54	0.874	90	1.515	0.133
Secretary assistance	2.25	1.056	2.08	0.987	90	0.809	0.421
E-platform	2.13	1.305	2.21	0.871	90	−0.381	0.704
Technical services	1.95	1.131	2.04	0.969	90	−0.403	0.688
Teaching quality	3.55	1.061	2.94	1.037	90	2.759	<0.01
Teachers’ knowledge	3.83	0.931	3.35	1.046	90	2.283	<0.05
University notes	3.30	1.067	2.79	1.073	90	2.273	<0.05
Testing mode	3.58	0.874	2.52	1.000	90	5.300	<0.001
Grading mode	3.43	1.059	2.54	1.056	90	3.985	<0.001

**Table 3:** Descriptive statistics for DASS-21 depression, anxiety, and stress in the two experimental subgroups, the mechanical and civil engineers, and the control group, the electrical engineers.

DASS-21 Factors	Departments	M	SD
Depression Pre	Electrical	0.503	0.629
	Mechanical	0.857	0.383
	Civil	0.908	0.766
Depression Mid	Electrical	0.725	0.611
	Mechanical	0.478	0.396
	Civil	0.672	0.717
Depression Post	Electrical	0.824	0.612
	Mechanical	0.391	0.398
	Civil	0.429	0.505
Anxiety Pre	Electrical	0.352	0.503
	Mechanical	0.547	0.394
	Civil	0.538	0.514
Anxiety Mid	Electrical	0.519	0.540
	Mechanical	0.335	0.403
	Civil	0.513	0.503
Anxiety Post	Electrical	0.563	0.587
	Mechanical	0.329	0.427
	Civil	0.454	0.423
Stress Pre	Electrical	0.599	0.557
	Mechanical	1.199	0.604
	Civil	1.445	0.654
Stress Mid	Electrical	0.813	0.623
	Mechanical	0.932	0.489
	Civil	1.109	0.588
Stress Post	Electrical	0.898	0.658
	Mechanical	0.609	0.476
	Civil	0.790	0.438

Note: Pre—previous, Mid—middle, and Post—posterior.

## Data Availability

The data presented in this study are available on request from the corresponding author due to privacy and ethical reasons.
